# Supplementary material for: Calendula officinalis Triterpenoid Saponins Impact the Immune Recognition of Proteins in Parasitic Nematodes
Source: Pathogens. 2021 Mar 4;10(3):296. doi: 10.3390/pathogens10030296 (PMC7999767; doi:10.3390/pathogens10030296)
Supplement: Supplementary file 1 [file pathogens-10-00296-s001.zip › pathogens-1109422-supplementary materials-final/Pathogens_Supplementary Dataset_Figure S.docx]

**Figure S.** *Calendula officinalis* triterpenoid saponins impact the immune recognition of *Heligmosomoides polygyrus bakeri* L3 larvae proteins. SDS-PAGE protein pattern of *H. polygyrus bakeri* L3- (**A**) and Western blot protein pattern of L3 recognized by hyperimmunne rabbit serum, immunized with L3(CTR)- (**B**). Gel slots loaded with 10 μg nematode protein. For band separation each slot was loaded with 30 μg nematode protein; expanded immunogram is shown (**C**). Proteins extracted from: 1 - control larvae L3(CTR), 2 - larvae exposed to 8% ethanol L3(EtOH), 3 - larvae exposed to *C. officinalis* glucuronides L3(GlcUAOA): kDa – molecular mass of protein marker.

**
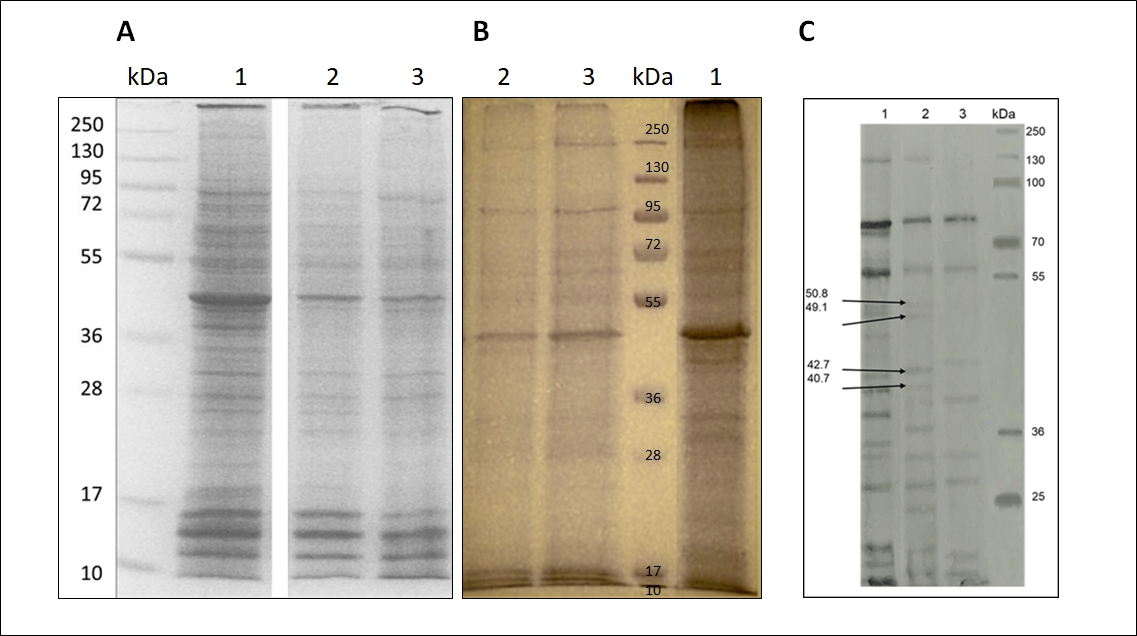
**
